# Profiling the TRB and IGH repertoire of patients with H5N6 Avian Influenza Virus Infection by high-throughput sequencing

**DOI:** 10.1038/s41598-019-43648-y

**Published:** 2019-05-15

**Authors:** Wujian Peng, Song Liu, Jingye Meng, Jiali Huang, Jianrong Huang, Donge Tang, Yong Dai

**Affiliations:** 1 The Third People’s Hospital of Shenzhen (The Affiliated Shenzhen Third Hospital, Guangdong Medical College), Shenzhen, Guangdong 518112 P.R. China; 2 Clinical medical research center, The Second Clinical Medical College of Jinan University (Shenzhen People’s Hospital), Shenzhen, Guangdong 518020 P.R. China; 3 Zhaoqing First People’s Hospital, Zhaoqing, 526020 China

**Keywords:** Genetics, Immunology

## Abstract

Avian Influenza A (H5N6) Virus causes severe influenza disease in humans and is manifested by acute respiratory distress syndrome, multi-organ failure, and high mortality rates. T cells recognize antigens specifically through a membrane protein T cell receptor (TCR). To ward off a wide variety of pathogens, the human adaptive immune system harbors a vast array of TCRs, which are collectively referred to as the TCR repertoire. The B cell receptor (BCR) is involved in inducing the humoral immune response. The generation of a diverse T cell and B cell repertoire is essential for protection against infection. In this study, multiplex PCR based on genomic DNA amplicons and Illumina high-throughput sequencing (HTS) were applied to study the characteristics and polymorphisms of the TRB and IGH repertoire in the peripheral blood mononuclear cells (PBMCs) from two H5N6 AIV patients and six healthy donors (NC). The CDR3 average length in the AIV group was different from the NC group. The TRBV12-3, TRBV12-4, and TRBV15 gene segments and TRBV30/TRBJ1-2, TRBV12-3/TRBJ1-1 and IGHV3-11/IGHJ6 gene segment pairings also exhibited a higher usage in the PBMCs of AIV donors and may provide more information for generating more effective T/B cell targeted diagnosis/protection strategies.

## Introduction

Avian influenza viruses (AIVs) circulate naturally in wild aquatic birds, infect domestic poultry, and are capable of causing sporadic bird-to-human transmissions. Highly pathogenic influenza A viruses are endemic to many countries, cause tremendous economic losses to the poultry industry, and represent a serious threat to public health. Genetic studies show that the H5N6 virus results from an exchange of genes from H5N1 viruses and H6N6 viruses that widely circulate in ducks. Since the new strain of avian influenza A virus (H5N6) was isolated from a patient^1^, some cases of H5N6 avian influenza infection were identified in the Sichuan (May, 2014)^1^, Guangdong (November, 2014)^2^ and Yunnan provinces (2015)^3^ in China. In January 2016, we encountered two laboratory-confirmed cases of human infection with avian influenza A (H5N6) virus by the National Health and Family Planning Commission (NHFPC) of China. The patients exhibited upper respiratory catarrh symptoms, aryngalgia, a high fever and hypodynamia in the early stages of the disease.

Avian influenza viruses have inherently different pathogeneses. For example, AIVs can be divided into non-pathogenic AIVs (NPAIV), low pathogenic AIVs (LPAIV), and highly pathogenic AIVs (HPAIV). H5N6 AIV is constantly evolving, and as such, novel AIVs possessing H5- and H6-derived internal genes and other AIVs possessing specific mammal-derived mutations could enhance its virulence and transmissibility in humans^2^. After an acute response to a viral infection, viral antigen (Ag) specific effector CD8^+^T cell clones are activated, which accelerate viral clearance and eliminate infected host cells^4^. Influenza virus-specific T cell clones are determined by the T cell receptor (TCR), which is composed of α- and β- chains. The structural diversity of the TCR α-and β- chains is generated by random recombination of the variable (*V*), diversity (*D*), joining (*J*), and constant (*C*) genes^5^. Theoretical TCR diversity in humans is placed in the region of 10^15^–10^20^ unique structures^6^, with direct *in vivo* estimates in greater than 2.5 × 10^7^ unique structures^7^. Lawson *et al*. demonstrated that most adults have circulating influenza-specific memory CD8^+^ T cells^8^, which provide protection against pursuant virus exposure^9^. After the Influenza virus is cleared, antigen specific memory CD8^+^ T cells are long-lived and are capable of rapid recall to effector function. Evidence^10,11^ suggests that CD8^+^ T-cells mediate cross-reactive protection in the face of heterologous prime/challenge among H1N1, H2N2, H3N2 and H5N1 viruses. However, neutralizing antibodies generated against HA can protect from infection with a specific strain, and these antibody responses are generally not cross-reactive with other influenza strains^12^.

The adaptive immune system protects each individual against insults, such as infection, and responds to vaccination with B cell proliferation in response to the antigenic stimulation, which is composed of B and T cells and produces a large number of antibodies. The foundation of the adaptive immune response is based on the enormous diversity of T and B cells, which produce a large number of antibodies. This profound diversity of T (TCRs) and B cell receptors (BCRs) is generated by V–D–J gene recombination of the TCR/BCR locus and the subsequent somatic hypermutation and class-switching recombination of B cells after antigen stimulation. Understanding which B cells produce specific antibodies and their kinetics conferring immunity against the variety of antigens has application in the field of vaccine development and in assessing response to infection^13^. The diversity of the human B cell repertoire is estimated, and the theoretical potential includes up to 10^11^ unique variants^14^; such diversity makes it difficult to analyze the repertoire.

Next-generation sequencing technologies have opened a new era in the field of T/BCR repertoire research, which includes studies on immune repertoire reconstitution after therapy, response to vaccines and subpopulation repertoire structure^15^. Thus, the study of the immune repertoire, portrayed as the antigen-specific information within lymphocytes, is key to understanding the response of adaptive immunity during infection.

The present study showed the TCR and BCR sequence characteristics from the peripheral blood of two patients with H5N6 Avian Influenza Virus Infection by HTS. From the HTS findings, we searched potential reactive clones and offer important new information for the disease.

## Results

### Case report

The first case is a 40-year-old female from the Duanzhou District, Zhaoqing City, with an onset date of 22 December. The patient was admitted to the hospital on 28 December and is now in critical condition. The second case is a 25-year-old male from Shenzhen City, Guangdong Province, who developed symptoms on 1 January. The patient was admitted to the hospital on 4 January and is now cured and left the hospital. He has a history of visiting a live poultry market. He worked in his father’s restaurant and had no other medical problems in his past history.

The two patient’s conditions deteriorated rapidly, and one-sided pneumonia progressed to two-sided pneumonia. When patient 1 arrived at the designated hospital, her condition was critical. She was given invasive mechanical ventilation and was transferred to the intensive care unit immediately. Other treatments, including gastric protective treatments, methylprednisolone, intravenous immunoglobulin and fluid infusion, were given. The complications and treatments are shown in Table 1. Despite active treatment, her condition continued to deteriorate. Fifty days after admission, she died from multi-organ failure. The male patient arrived at the designated hospital with 80% oxygen saturation, and then, he received noninvasive ventilator-assisted breathing. He was also given treatments, including gastric protective treatments, methylprednisolone, intravenous immunoglobulin and fluid infusion. His condition improved gradually, and 14 days after the illness onset, he was discharged.

**Table 1:** Patient characteristics.

Characteristics	Patient 1	Patient 2	Normal value
Age	40	25	
Gender	Female	Male	
Exposure history to birds in the past 7 days	NO	No	
Underlying condition	NO	No	
**Symptom**			
Fever	42	39.5	
Cough	Yes	Yes	
expectoration		Yes	
Cough with blood tinged sputum	Yes	Yes	
Sore throat	No	No	
dizziness	No	No	
headache	Yes	Yes	
myalgia	No	No	
Shortness of breath	Yes	Yes	
dyspnea	Yes	Yes	
Chest pain	Yes	Yes	
diarrhea	No	No	
nausea	No	No	
vomiting	No	No	
Skin ecchymosis	No	No	
coma	No	No	
**Blood cell count**			
**Blood gas analysis**			
PH	7.26	7.477	7.35–7.45
PO_2_	50	37.2	75–110
PCO_2_	41	39.7	35–45
SPO_2_	81	80%	90–100%
**complications**			
Septic shock	Yes	No	
Respiratory	Yes	Yes	
Acute respiratory distress syndrome	Yes	Yes	
Acute renal damage	No	No	
Multiple organ failure	Yes	No	
Diffuse intravascular coagulation	No	No	
Secondary infections	Yes	Yes	
**Treatment**			
Oxygen therapy	Yes	Yes	
continuous renal replacement	Yes	No	
Antibiotic therapy	Imipenem Vancomycin Moxifloxacin	Moxifloxacin	
Antiviral agent	Oseltamivir oral 150 mg bid Peramivir Intravenous 0.3 qd、Zanamivir Inhalation 10 mg bid	Oseltamivir oral 150 mg bid Peramivir、Intravenous 0.3 qd Zanamivir Inhalation 10 mg bid	
Glucocorticoid therapy	Methylprednisolone 80 mg Q12h	Methylprednisolone 60 mg Q12h	
Intravenous immunoglobulin therapy	10 g	15 g	
Mechanical ventilation	invasive mechanical ventilation	noninvasive ventilator-assisted breathing	
**Lab Test**			
WBC (×10^9^/L)	7.05	7.8	3.5–9.5
Neutrophils (×10^9^/L)	6.07	6.76	1.8–6.3
Lymphocytes (×10^9^/L)	0.81	0.79	1.1–3.2
CRP (mg/L)	>200.0	89.53	<8
ALT (U/L)	56	22	0–45
AST (U/L)	53	38	0–45
LDH (U/L)	78	532	120–250
CK (U/L)	276	1082	50–310
PCT(ng/mL)	25.04	0.333	0

### Summary of sequencing

To investigate the characteristics of the T/B cell immunity of the patient, the TCR β chains and BCR IGH from the PBMCs were sequenced using a HTS platform (IlluminaMiseq). The CDR3 sequences are identified by the conserved motif, and the abundance of each CDR3 clone and the number of distinct CDR3 clone species were calculated. The raw data are shown in Table 2. The profiles of the CDR3 lengths are an important determinant of the T/B cell repertoire diversity and were consistent with a polyclonal representation of clonotypic TCR and BCR sequences. The TCR CDR3 length varied from 6 to 19 nt, with a peak at 11 or 12 nt. The BCR CDR3 length varied from 4 to 29 nt, with a peak at 11, 16 or 17 nt. All the groups displayed good Gaussian distribution of the CDR3 length. Then, we fitted the Gaussian distribution curve for each sample with Excel, and the goodness of fit was quantified by R^2^, which ranged from 0 to 1 (that is, from worst fitness to best fitness).

**Table 2:** Statistics of the TCR and BCR sequences.

Items	Patient 1		Patient 2		Health control	
	TCR	BCR	TCR	BCR	TCR	BCR
Total reads number	632248	486372	709147	565148	1078038	466662
immune sequences number	625772	478308	702605	556630	1069580	462368
Unknown sequences number	6476	8064	6542	8518	8458	4294
productive sequences number	445475	376731	522948	457868	809132	361462
Non_productive sequences number	180297	101577	179657	98762	260448	100906
In-frame sequences number	464924	400538	549989	483511	841639	385295
Out-of_frame sequences number	157489	77377	149177	72843	223826	76805
Total CDR3 sequences number	431848	354183	513044	428262	795732	349989
Unique cdr3 nt sequences number	23920	18066	21255	20420	46012	32346
Unique cdr3 aa sequences number	19120	12554	16783	14287	37531	24048
Highly expended clone number all	1	36	8	33	6	0
Highly expended clone ratio all	0.006097053	0.253643	0.108823025	0.247577	0.077371527	0
Shannon entropy all	0.599300084	0.515524	0.519455186	0.50069	0.601856778	0.656329

### TRBV and TRBJ usage

**Figure 1:**
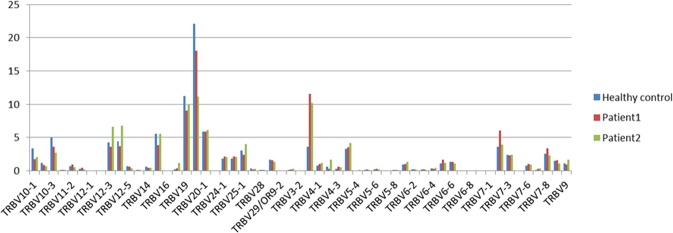
TRBV segments usage ratio in patients and Health control. Relative frequency of TRBV segments for the set of all clonotypes; Purple bars represented for the patient 1, red bars represented for patient 1, and yellow bars for health controls (n = 6). Usage ratio of TRBV genes ranged from 0.00% of TRBV6-8 to 11.1% of TRBV2 in the patient 1, while the usage ratio ranged from 0.01% of TRBV3-2 to 11.52% of TRBV30 in the patient 2.

**Figure 2:**
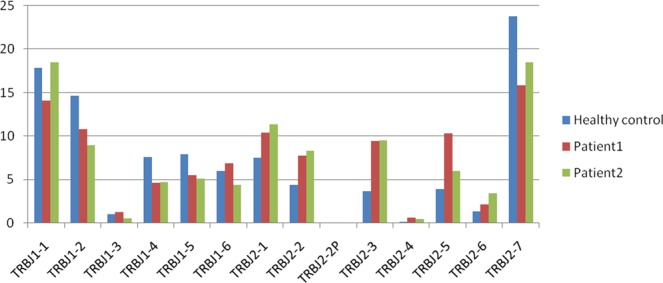
TRBJ segments usage ratio in patients and Health control. Usage ratio of this set of 14 TRBV genes ranged from 0.45% of TRBJ2-4 to 18.54% of TRBJ1-1in the patient 2. which had similar ratio in patient 2 and control.

The IMGT TRB gene database collected 48 functional TRBV genes and 13 functional TRBJ genes^16,17^. Here, 57 distinct TRBV genes and 14 distinct TRBJ genes were detected. The usage ratio of this set of 57 TRBV genes ranged from 0.00% of TRBV6-8 to 11.1% of TRBV2 in patient 1, while the usage ratio ranged from 0.01% of TRBV3-2 to 11.52% of TRBV30 in patient 2 (Fig. 1). The gene expression level of TRBV2 was the highest in both the patients and healthy controls. TRBV30 was highly expressed in the two patients (10.15%, 11.52%) but was low in the controls (3.62%). TRBV7-2 showed the highest expression in patient 1, while TRBV12-3, TRBV12-4 and TRBV15 showed the opposite. The data suggested that these genes might be associated with the severity of the disease, which may provide protection. The usage ratio of this set of 14 TRBJ genes ranged from 0.45% of TRBJ2-4 to 18.54% of TRBJ1-1 in patient 2 and showed a similar ratio in patient 2 and the controls (Fig. 2).

In the present study, 598, 598 and 626 TRBV-TRBJ pairings were identified in the two patients and controls. The significant pairs are listed (Table 3). The most significant pairing from patient 1 was TRBV30/TRBJ1-2, accounting for 2.61% of the total pairings, while the most frequent pairing from patient 2 was TRBV12-3/TRBJ1-1, accounting for 8.04% of the total pairings.

**Table 3:** The top twenty TRBV–TRBJ pairs.

Patient 1			Patient 2			Health control		
V_type	J_type	Percent	V_type	J_type	Percent	V_type	J_type	Percent
TRBV30	TRBJ1-2	2.6111039	TRBV12-3	TRBJ1-1	8.0410257	TRBV2	TRBJ1-2	5.6067872
TRBV30	TRBJ1-1	2.4110335	TRBV2	TRBJ2-7	1.9604556	TRBV2	TRBJ2-7	4.9607154
TRBV2	TRBJ2-7	2.322808	TRBV2	TRBJ1-2	1.8949642	TRBV19	TRBJ1-1	3.4432447
TRBV2	TRBJ2-5	2.1509883	TRBV30	TRBJ2-7	1.8325914	TRBV2	TRBJ1-1	2.9032388
TRBV2	TRBJ2-3	2.0949501	TRBV19	TRBJ1-1	1.789905	TRBV2	TRBJ1-4	2.6417186
TRBV30	TRBJ2-7	1.9527704	TRBV30	TRBJ1-1	1.7606677	TRBV12-3	TRBJ2-7	2.2545279
TRBV2	TRBJ2-1	1.870334	TRBV2	TRBJ2-1	1.7355237	TRBV19	TRBJ2-7	2.0221632
TRBV2	TRBJ1-4	1.781877	TRBV15	TRBJ2-7	1.6181848	TRBV2	TRBJ1-5	1.9191135
TRBV2	TRBJ2-2	1.7225968	TRBV19	TRBJ2-7	1.4879815	TRBV12-3	TRBJ1-2	1.8680913
TRBV2	TRBJ1-1	1.7202812	TRBV19	TRBJ2-2	1.3519308	TRBV20-1	TRBJ2-7	1.683607
TRBV19	TRBJ1-1	1.4780664	TRBV12-3	TRBJ2-7	1.30788	TRBV10-3	TRBJ2-5	1.5929986
TRBV2	TRBJ1-2	1.4345325	TRBV20-1	TRBJ2-7	1.2585665	TRBV15	TRBJ2-7	1.4333972
TRBV7-2	TRBJ2-7	1.3995665	TRBV25-1	TRBJ1-6	1.2375157	TRBV19	TRBJ1-5	1.4238462
TRBV12-3	TRBJ1-1	1.3613586	TRBV19	TRBJ2-1	1.2328377	TRBV12-3	TRBJ1-1	1.3305987
TRBV19	TRBJ1-5	1.3602008	TRBV2	TRBJ2-6	1.2283547	TRBV19	TRBJ1-2	1.1632057
TRBV2	TRBJ1-6	1.2944369	TRBV7-2	TRBJ2-7	1.2137361	TRBV2	TRBJ2-1	1.119221
TRBV24-1	TRBJ2-1	1.27267	TRBV20-1	TRBJ2-1	1.1435666	TRBV10-1	TRBJ2-7	1.1121835
TRBV12-3	TRBJ2-7	1.2082955	TRBV30	TRBJ2-2	1.126609	TRBV2	TRBJ1-6	1.095218
TRBV12-3	TRBJ2-2	1.1325744	TRBV24-1	TRBJ1-2	1.1057531	TRBV7-2	TRBJ2-7	1.063926
TRBV19	TRBJ2-7	1.0723681	TRBV5-1	TRBJ2-7	1.1022446	TRBV10-1	TRBJ1-1	1.0454525

### TRBV CDR3 sequence diversity

The extent of the interindividual TCR sharing was determined by the TCR clonotype frequencies. Our analysis focused mainly on the DNA and aa sequences of complementarity-determining region 3 (CDR3), which is the most diverse region of the TCR molecule and is associated with antigen epitope recognition. In this study, we defined that clones with a frequency above 0.1% of total reads in a sample were Highly expanded clones (HECs). There were 9 TCR highly expressed clones in the patients. A summary of these HEC sequences is presented in Table 4. Next, we investigated whether these HECs overlapped between the different patients, but we found that patient 2 shared some HEC sequences with the controls. Patient 1 was critical, which means that the HEC may be associated with the severity of AIV.

**Table 4:** A list of the public TCR CDR3 sequences.

Clonotype	Public sequence	P value		
		Patient 1	Patient 2	Health control
DNA	GCCAGCACGGGCGGCTATGGCTACACC	9.26E-05	0.00640101	0.006493636
	GCCAGCAGCCAGGGCGGATTTAATTCACCCCTCCAC	9.73E-05	0.008936855	0.009034112
	GCCAGCAGCCTTCAGGGGCGGACTGAAGCTTTC	0.000694689	0.059127482	0.059822171
	GCCAGCAGCTCCTCCGGGACTATTAACTACGAGCAGTAC	0.000120413	0.008946601	0.009067014
	GCCAGCAGTAATGGACGGCCTAATGAAAAACTGTTT	7.87E-05	0.005746096	0.005824827
	GCCAGCTCACCACCGAGACTAGCGGACACCGGGGAGCTGTTT	9.49E-05	0.005555079	0.00565002
	GCCAGTAACTCAGGCGGGGAGCTGTTT	8.34E-05	0.007677704	0.007761067
	GCCAGTAGTCCTCGACACGGACAACCGAACACTGAAGCTTTC	9.03E-05	0.006432197	0.006522506
	GCCTGGAGTTCCTCGGGACCGACCGACGAGCAGTAC	0.006097053	1.17E-05	0.006432197
AA	ASNSGGELF	8.57E-05	0.007728382	0.00781406
	ASSLQGRTEAF	0.000708583	0.060086464	0.060795047
	ASSNGRPNEKLF	7.87E-05	0.005820164	0.005898895
	ASSPPRLADTGELF	9.73E-05	0.005666181	0.005763437
	ASSPRHGQPNTEAF	9.03E-05	0.006576434	0.006666744
	ASSQGGFNSPLH	9.73E-05	0.009084991	0.009182247
	ASSSSGTINYEQY	0.000122728	0.009055754	0.009178482
	ASTGGYGYT	9.49E-05	0.006457536	0.006552477

### IGHD, IGHV and IGHJ usage

**Figure 3:**
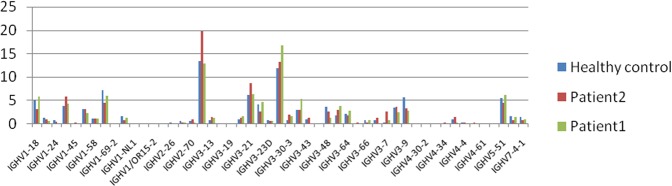
IGHV segments usage ratio in patients and Health control. Bars indicate the respective percentages of the results from patients and health group.

**Figure 4:**
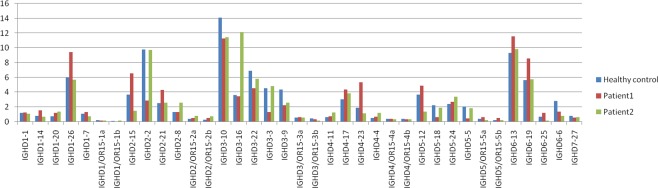
IGHD segments usage ratio in patients and Health control. Bars indicate the respective percentages of the results from patients and health group.

To evaluate the change in the IGH gene frequency of the AIV patients, we calculated the frequency of the IGHV, IGHD and IGHJ gene subgroups of two AIV patients. Ninety IGHV segments, 37 IGHD segments and 6 IGHJ segments were all included. Some of the IGHVs were not detected in all the samples, such as IGHV1-38-4, IGHV1-68, IGHV1-69D, IGHV1/OR15-5, IGHV1/OR15-9, IGHV1/OR21-1, IGHV2/OR16-5, IGHV3-22, IGHV3-25, IGHV3-38-3, IGHV3-62, IGHV3- 71, IGHV3-72, IGHV3-73, IGHV3/OR16-12, IGHV3/OR16-13, IGHV3/OR16-14, IGHV3/OR16-15, IGHV3/OR16-16, IGHV3/OR16-6, IGHV3/OR16-8, IGHV4-30-4 and IGHV7-81 (Fig. 3). For the 37 IGHD segments, some of the IGHD segments were expressed higher in the female patient, such as IGHD6-13, IGHD1-26, IGHD6-19, IGHD2-15, IGHD4-23, and IGHD5-5, and IGHD5-18 had a low expression (Fig. 4).

In the present study, there were 301, 308 and 345 IGHV-IGHJ pairings identified in the two patients and the controls. The significant pairs are listed (Table 5). The most significant paring for patient 2 was IGHV3-11/IGHJ6, accounting for 20.09938% of the total pairings.

**Table 5:** The top twenty IGH V–IGH J pairs.

Patient 1			Patient 2			Health control		
V_type	J_type	Percent	V_type	J_type	Percent	V_type	J_type	Percent
IGHV3-11	IGHJ6	10.73541	IGHV3-11	IGHJ6	20.09938	IGHV3-11	IGHJ6	9.223718
IGHV3-30	IGHJ6	9.91719	IGHV3-30	IGHJ6	9.756878	IGHV3-30	IGHJ6	6.733354
IGHV3-30	IGHJ4	6.546333	IGHV3-11	IGHJ4	5.145448	IGHV3-11	IGHJ4	6.047619
IGHV3-11	IGHJ4	4.887869	IGHV3-30	IGHJ4	4.061766	IGHV3-30	IGHJ4	4.547857
IGHV5-51	IGHJ6	3.345728	IGHV3-11	IGHJ3	3.928436	IGHV1-69	IGHJ6	3.682116
IGHV1-69	IGHJ6	3.178018	IGHV1-3	IGHJ6	3.815888	IGHV3-11	IGHJ3	2.75923
IGHV3-33	IGHJ6	2.589622	IGHV5-51	IGHJ6	2.864602	IGHV3-30	IGHJ3	1.998063
IGHV3-53	IGHJ6	2.341163	IGHV1-69	IGHJ6	2.358369	IGHV1-18	IGHJ6	1.992348
IGHV3-11	IGHJ1	2.130537	IGHV3-74	IGHJ6	2.327547	IGHV3-23	IGHJ4	1.955776
IGHV3-30	IGHJ3	2.015907	IGHV1-18	IGHJ6	2.057852	IGHV1-3	IGHJ6	1.887774
IGHV1-18	IGHJ1	2.014495	IGHV3-30	IGHJ3	1.787924	IGHV3-9	IGHJ6	1.857773
IGHV3-23	IGHJ4	2.004613	IGHV3-53	IGHJ6	1.782787	IGHV3-23	IGHJ6	1.842058
IGHV5-51	IGHJ4	1.827304	IGHV3-9	IGHJ6	1.621204	IGHV1-69	IGHJ4	1.651195
IGHV1-3	IGHJ4	1.75446	IGHV1-46	IGHJ6	1.550686	IGHV5-51	IGHJ6	1.63548
IGHV1-3	IGHJ6	1.712109	IGHV3-48	IGHJ6	1.544849	IGHV5-51	IGHJ4	1.546049
IGHV1-18	IGHJ6	1.692345	IGHV3-11	IGHJ2	1.448412	IGHV1-18	IGHJ4	1.517762
IGHV3-9	IGHJ6	1.593809	IGHV3-23	IGHJ6	1.107033	IGHV3-48	IGHJ6	1.490333
IGHV3-11	IGHJ3	1.592679	IGHV3-43	IGHJ6	1.104698	IGHV3-9	IGHJ4	1.452903
IGHV1-18	IGHJ4	1.502331	IGHV1-69	IGHJ4	1.093022	IGHV3-11	IGHJ2	1.301184
IGHV1-69	IGHJ4	1.487084	IGHV3-11	IGHJ5	1.06687	IGHV1-46	IGHJ6	1.254039

### Highly expanded clones (HEC) and BCR repertoire diversity

The expression level of each clone was calculated according to the identity of each sequence after the alignment. The degree of expansion of each sample clone was based on the unique CDR3 sequence frequency. Here, we defined BCR clones with a frequency above 0.1% of the total reads in a sample as HECs. In the patient samples, we observed 36 clones in patient 1 and 33 in patient 2, while in the healthy control group, no HECs were defined. A comparison of the degree of expansion of the most expanded clones in each group showed that the HEC number and the HEC ratio in the AIV group were more expanded than those in the NC group. The shared B cells or public B cells are of long-term interest both in healthy and disease states. Next, we investigated whether the HECs overlapped between the different individuals of the same group or between the AIV and NC groups. We found that each individual had a unique repertoire, and no HEC was shared by different individuals. Our results suggest that the entire BCR repertoire of the peripheral blood has a much more skewed clonotype composition in AIV patients than in the healthy controls (Table 6).

**Table 6:** A list of the public BCR CDR3 sequences.

Clonotype	Public sequence	P value		
		Patient 1	Patient 2	Health control
DNA	ATGGACGTC	0.003391	1.40E-05	2.86E-06
	GCGAAAGATGTCCTTAGTGGGAGCTACTTAGACTACTACGGTATGGACGTC	2.82E-06	2.34E-06	7.71E-05
	GGTATGGACGTC	5.65E-06	2.34E-06	5.71E-06
AA	AKDVLSGSYLDYYGMDV	0.003391	2.34E-06	7.71E-05
	GMDV	2.82E-06	2.34E-06	5.71E-06
	MDV	5.65E-06	1.40E-05	2.86E-06

## Discussion

Humans H5N6 avian influenza viruses were first confirmed in China in May 2014. The patients all had direct contact with poultry prior to the onset of the disease^1,18^. However, these patients did not contact poultry, and they were infected with H5N6 influenza. Hence, indirect contact with poultry, such as passing a live poultry market, needs further attention. The two cases were adults aged 40 and 25 years. The clinical manifestations of these patients were unresolved fever, cough, expectoration of sputum, sometimes accompanied by hemoptysis, and shortness of breath. They occurred with a rapid progression of acute respiratory distress syndrome. These were consistent with the previous report of H5N1 infection^19^.

T lymphocytes play an important role in mediating anti-Influenza virus immune responses. Influenza virus can be cleared efficiently by the adaptive immune system, consisting of antibody-producing B cells and influenza-specific T lymphocytes with diverse functions. For mild infections, whether caused by seasonal influenza viruses or occasional asymptomatic AIVs, the memory CD8^+^ and/or CD4^+^ T cells provide a great level of protection^20,21^. The identification of human T cell epitopes helps in efforts to determine how concurrent simultaneous infection with H5N6 viruses affects CD8 T cell immunity. The treatment strategies will have to be modified to recruit low-affinity and cross-reactive T cells.

In the past, the study of the T/BCR repertoire was based on traditional technologies, such as polymerase chain reaction (PCR)-single strand conformation polymorphism analysis (SSCP), flow cytometry and immune spectratyping. The T/BCR repertoire is hard to reflect and is limited by the number of sequence data. A deep-sequencing approach to assess the T/BCR repertoire is possible with the advances in HTS, and we now have powerful tools to reveal information on the TCR and BCR repertoire after infectious states. Conversely, H5N6 avian influenza viruses-specific TCR/BCR clonalities will be valuable markers to assess the nature and quality of a vaccine response^22^.

In the present study, we used HTS of the human TCR/BCR heavy chain repertoire to gain insight into T/B-cell dynamics after H5N6 infection, and we obtained H5N6 infection-induced changes in the total repertoire. Direct sequencing of the TRBV CDR3 regions from the T-cell gDNA library using HTS fully captured all the CDR3 length diversity. Then, virtual TRBV spectra types, according to the CDR3 lengths of the 46 TRBV gene segments, were examined. In the Patients, V families (TRBV7-2, TRBV10-3, TRBV7-3, TRBV7-8, TRBV10-1, TRBV24-1, TRBV24/OR9-2) were demonstrated at the top. However, more V families (TRBV11-1, TRBV5-8, TRBV28, TRBV13, TRBV5-4, TRBV6-7, TRBV7-4, TRBV6-9, TRBV16, TRBV3-2) showed low expansion. However, TRBV4-2 was more highly expressed in patient 2 compared to the others. To better understand the shared nature of the T cells in disease presentation associated with H5N6 AIV infections, we analyzed the CDR3 of the clonally expanded T cells and found differentially expressed ASNSGGELF, ASSLQGRTEAF, ASSNGRPNEKLF, ASSPPRLADTGELF, ASSPR and HGQPNTEAF amino acid sequences in the CDR3 of the TRBV chains. These findings may facilitate the allogeneic adoptive T cell immunotherapy, and more dominant CDR3 sequences in the distinct length forming the clonal expansion would be easily identified by the high-through sequencing technology. Thus, the sequence information of the CDR3 may be a valuable resource for disease diagnosis and drug design.

The random assortment of the V, (D), and J gene segments provides the basic structural frame for the antibody variable region to recognize specific antigens. Until now, no experiments have studied the usage feature of the V, (D), and J gene segments in AIV. The BCR repertoires of the two AIV patients lost some IGHV usages in our study. We also found IGHV1-69-IGHJ6, IGHV1-18-IGHJ1, IGHV1-18-IGHJ6, IGHV3-30-IGHJ6, IGHV3-33-IGHJ6, IGHV3-11-IGHJ1, IGHV3-53-IGHJ6, IGH V3-74-IGHJ6, IGHV5- 51-IGHJ6, IGHV7-4-1-IGHJ5 and IGHV5-51-IGHJ6 pairings, which were dominant in the two AIV patients. These higher usage genes provide more information for generating a more effective B cell targeted therapy approach.

In addition, analyses of the composition of the TCR and H-CDR3 showed that the overall AA compositions of the CDR3 in each AIV patient were not significantly different. These results revealed that small numbers resulted in a lack of a validated system to describe patient outcome. While this is a descriptive and preliminary work in a very small group of patients, we used a novel NGS protocol to investigate the TRB and IGH repertoire of patients with H5N6 Avian Influenza Virus Infection at the sequence level for the first time. In further research, more samples will be included.

In conclusion, we demonstrated a successful approach for profiling the entire T/B cell repertoire at sequence-level resolution in AIV patients. By applying the method described herein, we identified some T/BCR repertoire features. These global repertories contain a wealth of information and provide us many opportunities to gain new insights into humoral immunity and offer practical results for the benefit of patients.

## Methods

### Patients and controls

Two cases of human infection with avian influenza A (H5N6) virus were laboratory-confirmed by the National Health and Family Planning Commission (NHFPC) of China. The first case is a 40-year-old female from the Duanzhou District, Zhaoqing City, with an onset date of 22 December. The second case is a 25-year-old male from Shenzhen City, Guangdong Province, who developed symptoms on 1 January. The 6 healthy controls were adults without any basic diseases, and they were three males (25–30 years old) and three females (35–40 years old). DNA from 6 control’ B cells and T cells were mixed together by 1:1:1:1:1:1 according to Qubit value, renamed one Heath control pool.

### Ethics statement

Written informed consent for study participation was obtained from all the subjects. The use of PBMCs for further studies beyond routine diagnosis was approved by the Third People’s Hospital of Shenzhen and the Zhaoqing First People’s Hospital ethics committee. This study abides by the Helsinki Declaration on ethical principles for medical research involving human subjects.

### Mononuclear cell preparation, T cell and B cell isolation and DNA extraction

Peripheral blood mononuclear cells (PBMCs) were prepared using fresh whole blood from 2 patients and 6 healthy individuals. The blood was collected in Monovette EDTA tubes. Cell isolations and DNA extractions were performed under Biosafety Level 3 containment. The PBMCs were isolated by density gradient centrifugation using Lymphoprep (Axis Shield, UK) according to the manufacturer’s instructions. B lymphocytes were isolated with MicroBeads (Miltenyi Biotech, Germany) according to the manufacturer’s instructions. Then, the B cells were enriched by magnetic cell sorting (MACS) according to the manufacturer’s instructions (Miltenyi Biotech). Non-B cells were indirectly magnetically labeled with a cocktail of biotin-conjugated monoclonal antibodies as the primary labeling reagent and anti-biotin monoclonal antibodies conjugated to MicroBeads as the secondary labeling reagent. The magnetically labeled non-B cells were depleted by retaining them on a MACS® Column in the magnetic field of a MACS Separator, while the unlabeled B cells passed through the column. The T cells were isolated with anti-human CD3 magnetic beads according to the manufacturer’s protocol (Miltenyi Biotec, Bergisch, Gladbach, Germany). The T-cell purity was >90% (data not shown), as determined by flow cytometry using mouse anti-human antibodies CD3-phycoerythrin (PE) (BD Biosciences, San Jose, CA). DNA was extracted using QIAamp DNA Mini Kit (Qiagen, Germany) following the manufacturer’s instructions, and the DNA integrity was analyzed by agarose gel electrophoresis.

### Primer design and multiplex-PCR amplification

The human IGH and TRB sequences were downloaded from IMGT (http://www.imgt.org/)^23^. A relative conserved region in frame region 3 upstream of CDR3 was selected for putative forward primer region. A cluster of primers corresponding to the majority of the V gene sequence family was selected. Similarly, reverse primers corresponding to 6 types of the J gene family were designed. The forward and reverse primers were analyzed by Oligo 7.0 and MFEprimer-2.0 for primer dimer and loop structures. Minor changes of the sequences were made for low-quality primers. The final IGH CDR3 primer sequences are shown in Table 7. The utilized 12 forward primers and 4 reverse primers were used for multiplex PCR to amplify rearranged IGH CDR3 region. Similarly, the utilized 30 forward primers and 13 reverse primers were used for multiplex PCR to amplify rearranged TCR-β CDR3 region (Table 8).

**Table 7:** Multiplex-PCR amplification primers of the IGH CDR3 region.

primers	sequences
IGHV1-18	CAGACGTGTGCTCTTCCGATCTAGAGAGTCACCATGACCACAGAC
IGHV1-2/1-46	CAGACGTGTGCTCTTCCGATCTAGAGAGTCACCAKKACCAGGGAC
IGHV1-24	CAGACGTGTGCTCTTCCGATCTAGAGAGTCACCATGACCGAGGAC
IGHV1-3/1-45	CAGACGTGTGCTCTTCCGATCTAGAGAGTCACCATTACYAGGGAC
IGHV1-69/1-f	CAGACGTGTGCTCTTCCGATCTAGAGAGTCACGATWACCRCGGAC
IGHV1-8	CAGACGTGTGCTCTTCCGATCTAGAGAGTCACCATGACCAGGAAC
IGH2-70/26/5	CAGACGTGTGCTCTTCCGATCTAGACCAGGCTCACCATYWCCAAGG
IGHV3	CAGACGTGTGCTCTTCCGATCTAGGGCCGATTCACCATCTCMAG
IGH4	CAGACGTGTGCTCTTCCGATCTAGCGAGTCACCATRTCMGTAGAC
IGHV5-51	CAGACGTGTGCTCTTCCGATCTAGCAGCCGACAAGTCCATCAGC
IGHV6-1	CAGACGTGTGCTCTTCCGATCTAGAGTCGAATAACCATCAACCCAG
IGHV7-NEW	CAGACGTGTGCTCTTCCGATCTAGGACGGTTTGTCTTCTCCTTG
HIGHJ-Rev1	CTACACGACGCTCTTCCGATCTCTGAGGAGACRGTGACCAGGGTG
HIGHJ-Rev2	CTACACGACGCTCTTCCGATCTCTGAAGAGACGGTGACCATTGTC
HIGHJ-Rev3	CTACACGACGCTCTTCCGATCTCTGAGGAGACGGTGACCAGGGT
HIGHJ-Rev4	CTACACGACGCTCTTCCGATCTTGAGGAGACGGTGACCGTGGTC

**Table 8:** Multiplex-PCR amplification primers of the TRB CDR3 region.

Primers	Sequences
TRBV2F-IND	CAGACGTGTGCTCTTCCGATCTAGATTTCACTCTGAAGATCCGGTCCAC
TRBV9F-IND	CAGACGTGTGCTCTTCCGATCTAGCCTGACTTGCACTCTGAACTAAACCT
TRBV14F-IND	CAGACGTGTGCTCTTCCGATCTAGGGAGGGACGTATTCTACTCTGAAGG
TRBV15F-IND	CAGACGTGTGCTCTTCCGATCTAGTTCTTGACATCCGCTCACCAGG
TRBV19F-IND	CAGACGTGTGCTCTTCCGATCTAGTCCTTTCCTCTCACTGTGACATCGG
TRBV3-1-F4-IND	CAGACGTGTGCTCTTCCGATCTAGAAACAGTTCCAAATCGMTTCTCAC
TRBV4-1/2/3-F4-IND	CAGACGTGTGCTCTTCCGATCTAGCAAGTCGCTTCTCACCTGAATG
TRBV5-1-F4-IND	CAGACGTGTGCTCTTCCGATCTAGGCCAGTTCTCTAACTCTCGCTCT
TRBV5-4/5/6/8-F4-IND	CAGACGTGTGCTCTTCCGATCTAGTCAGGTCGCCAGTTCCCTAAYTAT
TRBV6-1/2/3/5/8-F4-IND	CAGACGTGTGCTCTTCCGATCTAGCAATGGCTACAATGTCTCYAGAT
TRBV6-4-F4-IND	CAGACGTGTGCTCTTCCGATCTAGTGATGGTTATAGTGTCTCCAGAG
TRBV6-9-F4-IND	CAGACGTGTGCTCTTCCGATCTAGCGATGGCTACAATGTATCCAGAT
TRBV6-6-F4-IND	CAGACGTGTGCTCTTCCGATCTAGGAATGGCTACAACGTCTCCAGAT
TRBV7-2/4/6/7/8-F4-IND	CAGACGTGTGCTCTTCCGATCTAGGGGATCCGTCTCCACTCTGAMGAT
TRBV7-3-F4-IND	CAGACGTGTGCTCTTCCGATCTAGGGGATCCGTCTCTACTCTGAAGAT
TRBV7-9-F4-IND	CAGACGTGTGCTCTTCCGATCTAGGGGATCTTTCTCCACCTTGGAGAT
TRBV10-1-F4-IND	CAGACGTGTGCTCTTCCGATCTAGCCTCACTCTGGAGTCTGCTGCC
TRBV10-2/3-F4-IND	CAGACGTGTGCTCTTCCGATCTAGCCTCACTCTGGAGTCMGCTACC
TRBV11-1/2/3-F4-IND	CAGACGTGTGCTCTTCCGATCTAGGCAGAGAGGCTCAAAGGAGTAGACT
TRBV12-3/4-F4-IND	CAGACGTGTGCTCTTCCGATCTAGATCGATTCTCAGCTAAGATGCCT
TRBV12-5-F4-IND	CAGACGTGTGCTCTTCCGATCTAGATCGATTCTCAGCAGAGATGCCT
TRBV13-F4-IND	CAGACGTGTGCTCTTCCGATCTAGTCGATTCTCAGCTCAACAGTTC
TRBV18-F4-IND	CAGACGTGTGCTCTTCCGATCTAGTAGATGAGTCAGGAATGCCAAAG
TRBV20-1-F4-IND	CAGACGTGTGCTCTTCCGATCTAGAACCATGCAAGCCTGACCTT
TRBV24-1-F2-IND	CAGACGTGTGCTCTTCCGATCTAGCTCCCTGTCCCTAGAGTCTGCCAT
TRBV25-1F-IND	CAGACGTGTGCTCTTCCGATCTAGGCCCTCACATACCTCTCAGTACCTC
TRBV27/28-F4-IND	CAGACGTGTGCTCTTCCGATCTAGGGAGATGTTCCTGARGGGTACA
TRBV29-1-F4-IND	CAGACGTGTGCTCTTCCGATCTAGAACTCTGACTGTGAGCAACATGAG
TRBV16-F2-IND	CAGACGTGTGCTCTTCCGATCTAGCTGTAGCCTTGAGATCCAGGCTACGA
TRBV30-F5-IND	CAGACGTGTGCTCTTCCGATCTAGCAGATCAGCTCTGAGGTGCCCCA
TRBJ1.1-R2-P1	CTACACGACGCTCTTCCGATCTCTTACCTACAACTGTGAGTCTGGTG
TRBJ1.2R-P1	CTACACGACGCTCTTCCGATCTCTTACCTACAACGGTTAACCTGGTC
TRBJ1.3R-P1	CTACACGACGCTCTTCCGATCTCTTACCTACAACAGTGAGCCAACTT
TRBJ1.4R-P1	CTACACGACGCTCTTCCGATCTCATACCCAAGACAGAGAGCTGGGTTC
TRBJ1.5R-P1	CTACACGACGCTCTTCCGATCTCTTACCTAGGATGGAGAGTCGAGTC
TRBJ1.6R-P1	CTACACGACGCTCTTCCGATCTCATACCTGTCACAGTGAGCCTG
TRBJ2.1R-P1	CTACACGACGCTCTTCCGATCTCCTTCTTACCTAGCACGGTGA
TRBJ2.2R-P1	CTACACGACGCTCTTCCGATCTCTTACCCAGTACGGTCAGCCT
TRBJ2.3R-P1	CTACACGACGCTCTTCCGATCTCCGCTTACCGAGCACTGTCAG
TRBJ2.4R-P1	CTACACGACGCTCTTCCGATCTCCAGCTTACCCAGCACTGAGA
TRBJ2.5-R2-P1	CTACACGACGCTCTTCCGATCTCGAGCACCAGGAGCCGCGT
TRBJ2.6R-P1	CTACACGACGCTCTTCCGATCTCTCGCCCAGCACGGTCAGCCT
TRBJ2.7-R2-P1	CTACACGACGCTCTTCCGATCTCTTACCTGTGACCGTGAGCCTG

After amplification and agarose gel electrophoresis selection, the products were purified using the QIA quick PCR Purification Kit. The final library was quantitated in two ways as follows: by determining the average molecule length using the Agilent 2100 bioanalyzer instrument (Agilent DNA 1000 Reagents) and by real-time quantitative PCR (QPCR) (TaqMan Probe). The libraries were amplified with cBot to generate the cluster on the flow cell, and the amplified flow cell was pair-end sequenced using an Illumina Miseq instrument, with a read length of 150 bp as the most frequently used sequencing strategy.

The PCR conditions were set as 95 °C for 15 minutes, followed by 25 cycles of 94 °C for 15 seconds, 60 °C for 3 minutes, and a final extension for 10 minutes at 72 °C. The PCR products were purified by AMPure XP beads to remove the primer sequences (Beckman Coulter, Germany). A second round of PCR was performed to add a sequencing index to each sample. The PCR condition was set as 98 °C for 1 minutes, followed by 25 cycles of 98 °C for 20 seconds, 65 °C for 30 seconds and 72 °C for 30 seconds, with a final extension for 5 minutes at 72 °C. The library was separated on an agarose gel, and the target region was isolated and cleaned by QIAquick Gel Extraction Kits (Qiagen, Germany).

### HTS and data analysis

The PCR products were sequenced using an Illumina Genome Analyzer, and the sequencing quality of these read was evaluated by the formula shown below. The quality of the Miseq sequencing ranged from 0 to 40 and was used for filtering out low-quality reads. First, we filtered the raw data, including the adapter contamination. Reads with an average quality score lower than 15 (Illumina 0–41 quality system) were removed, and the proportion of N bases was not more than 5% (sequences with higher values were also removed). Next, a few bases with low quality (lower than 10) were trimmed; the quality score was expected to be over 15 after trimming, and the remaining sequence length was expected to be more than 60 nt. After filtering, the pair-end (PE) read pairs were merged into one contig sequence in two steps as follows: (1) by aligning the tail parts of two sequences and assessing the identity, with at least 10 bases of overlap required and the overlapping section having a 90% base match and (2) since different primers might result in sequences of different lengths, some might be very short (less than 100 bp) and will go through all the bases in the sequence, and thus, such reads were merged by aligning the head part of the sequence. In this way, we obtained the merged contig sequences and the length distribution plot. Subsequently, we used the Tcrip algorithm to perform the sequence analysis. The sequencing reads were mapped to the TCR and BCR sequences downloaded from IMGT/GENE-DB (http://www.imgt.org). The V, D, and J genes were designated according to the nomenclature provided by the international ImMunoGeneTics information system (IMGT). After alignment, we utilized the following method for the sequence structural analysis: (1) we calculated the number of each nucleotide and analyzed the proportion at each position; (2) according to the last position of the V gene, the start site of the D gene, the end site of the D gene, and the start site of the J gene after alignment, we retrieved the INDEL (insertion and deletion) introduced during V–D–J recombination; and (3) nucleotides were translated into amino acids. According to the identity of each sequence after the alignment, the expression level of each clone was clear and calculated. The expression of each distinct DNA sequence, amino acid sequence and V–J combination was also identified. In addition, to measure the diversity of each sample, we calculated the distinct clone number, Simpson coefficient and Shannon–Waver coefficient based on the different resolutions of the distinct DNA sequences, amino acid sequences, and V–J combinations. The expression level of each sample was also calculated at different resolutions of distinct DNA sequences, amino acid sequences, and V–J combinations. Moreover, we constructed the specific expression draft and plotted a heat-map according to the V–J combination profile. The diversity of the TCR and BCR repertoire was calculated based on the Simpson index of diversity (Ds)^24^ and the Shannon–Wiener index (H)^25^.

### Statistical analysis

The statistical analyses were conducted with GraphPad Prism software (GraphPad Software, San Diego, CA, USA). The p Values lower than 0.05 were considered significant.
